# Comparative Transcriptomic Analysis of *Riptortus pedestris* (Hemiptera: Alydidae) to Characterize Wing Formation across All Developmental Stages

**DOI:** 10.3390/insects12030226

**Published:** 2021-03-05

**Authors:** Siying Fu, Yujie Duan, Siqi Wang, Yipeng Ren, Wenjun Bu

**Affiliations:** Institute of Entomology, College of Life Sciences, Nankai University, Tianjin 300071, China; nkufsy@163.com (S.F.); yujieduan@mail.nankai.edu.cn (Y.D.); marshmallow311@163.com (S.W.)

**Keywords:** *Riptortus pedestris*, transcriptome library, wing formation, expression levels, pest control

## Abstract

**Simple Summary:**

*Riptortus pedestris* is a widely distributed pest insect in East Asia that causes considerable economic losses. In this study, we applied the Illumina HiSeq6000 platform to construct and sequence the transcriptome libraries of *R. pedestris* during all life stages. First, a total of 60,058 unigenes were assembled from raw data, and then annotated and classified with various databases. Furthermore, different numbers of differentially expressed genes were calculated by pairwise comparisons of all life stages, and some of these DEGs were associated with various functions by GO and KEGG analysis. Additionally, a total number of 35,158 SSRs and 715,604 SNPs were identified from all the transcriptome libraries. Finally, we analyzed ten wing formation-related signaling pathways, and detected the molecular and expression characterization of five wing development-related genes by qRT-PCR for all developmental stages of *R. pedestris*. Collectively, all these data may pave the avenue for exploring the developmental processes of hemimetabolous insects and pest management.

**Abstract:**

*Riptortus pedestris* (Hemiptera: Alydidae) is a major agricultural pest in East Asia that causes considerable economic losses to the soybean crop each year. However, the molecular mechanisms governing the growth and development of *R. pedestris* have not been fully elucidated. In this study, the Illumina HiSeq6000 platform was employed to perform de novo transcriptome assembly and determine the gene expression profiles of this species across all developmental stages, including eggs, first-, second-, third-, fourth-, and fifth-instar nymphs, and adults. In this study, a total of 60,058 unigenes were assembled from numerous raw reads, exhibiting an N50 length of 2126 bp and an average length of 1199 bp, and the unigenes were annotated and classified with various databases, such as the Kyoto Encyclopedia of Genes and Genomes (KEGG), Clusters of Orthologous Groups (COG), and Gene Ontology (GO). Furthermore, various numbers of differentially expressed genes (DEGs) were calculated through pairwise comparisons of all life stages, and some of these DEGs were associated with immunity, metabolism, and development by GO and KEGG enrichment. In addition, 35,158 simple sequence repeats (SSRs) and 715,604 potential single nucleotide polymorphisms (SNPs) were identified from the seven transcriptome libraries of *R. pedestris*. Finally, we identified and summarized ten wing formation-related signaling pathways, and the molecular properties and expression levels of five wing development-related genes were analyzed using quantitative real-time PCR for all developmental stages of *R. pedestris*. Taken together, the results of this study may establish a foundation for future research investigating developmental processes and wing formation in hemimetabolous insects and may provide valuable data for pest control efforts attempting to reduce the economic damage caused by this pest.

## 1. Introduction

The bean bug *Riptortus pedestris* (Fabricius 1775) is a polyphagous and widespread pest insect in a number of East Asian countries, including China, Japan, South Korea, and Thailand [1]. Both adults and nymphs of *R. pedestris* feed on leguminous plants or fruit trees, such as kidney bean (*Phaseolus vulgaris* L.) and apple (*Malus pumila* Miller), and transmit viruses or microorganisms, causing an increasing number of empty pods and undeveloped seeds based on the developmental stage and density of the insects [2]. In recent years, an increasing number of scientists have investigated the potential of this pest to cause damage to soybean in China, especially in the summer growing season from August to September [3]. However, there are many challenges to controlling the growth and development of *R. pedestris*. For example, the overuse of chemical insecticides can induce strong selection pressure on insect populations, favoring the emergence of resistant insects, which results in the alleviation of pesticidal effects and secondary influences on environmental safety and human health [4]. Therefore, it is urgently important to identify suitable methods of pest management that balance environmental safety and agricultural benefits.

Hemimetabolous insects, such as aphids, whiteflies, leafhoppers, and true bugs, undergo morphogenesis to produce mature external wings and genitalia during all their developmental stages, notably exhibiting crawler or nymph forms that appear similar to the adult forms [5]. In all these different types of metamorphosis, both holo- and hemimetabolous processes are regulated by different abiotic or biotic elements. In brief, various signaling pathways are activated by a series of receptors or factors involved in reproduction, development, diapauses, and metamorphosis, such as the TGF-β signaling pathway, Hippo signaling pathway, and insect hormone biosynthesis [6,7]. Furthermore, to respond to environmental changes, insects adopt several strategies, including altering their behavioral response by suppressing hormonal regulation or undergoing diapause. For instance, Ahn et al. described the simulated temperature-dependent adult emergence frequency and oviposition density curves of *R. pedestris* due to sensitivity to environmental conditions, suggesting that temperature is one of the most significant abiotic factors affecting the development, growth, and reproduction of insects. In addition, the females of *R. pedestris* undergo reproductive diapause after exposure to short-day (12 h light/12 h dark) photoperiods at 25 °C, whereas they start reproduction rearing long-day (16 h light/8 h dark) photoperiods within two weeks at the same temperature [2,8]. Collectively, these findings indicate that understanding the biotic or abiotic factors affecting pest development is essential to prevent and control *R. pedestris* infestations.

Over the past decade, advances in high-throughput next-generation sequencing (NGS) technology have made it possible to access large quantities of transcriptomic or genomic data in a cost-effective way [9]. De novo RNA sequencing and assembly have been successfully employed to retrieving genetic information (at the cell, tissue, or organism levels) at specific developmental stages or physiological conditions, including transcriptomic data from nonmodel species, by mapping known genomic data. Additionally, we also utilized NGS technology to estimate the gene expression changes under different developmental, metabolic, or pathogenic challenge conditions and identified a number of functional gene members without genome references [10]. To date, there have been few reports describing the comparative transcriptomic analysis of all developmental stages and molecular and expression characteristics of wing formation-related genes in *R. pedestris*, which could establish a foundation for future research seeking to prevent and control infestations of this economically important insect pest. In summary, multiomics techniques are powerful tools for elucidating the developmental processes of insects and may provide data for establishing management strategies for pest control.

In this research, we constructed and sequenced seven *R. pedestris* transcriptome libraries, including all developmental stages (eggs, 1st- to 5th-instar nymphs and adults), using a paired-end Illumina HiSeq6000 sequencing platform. First, clean reads, which were obtained by processing raw reads, were subjected to assembly and quality control to generate numerous unigenes, which were annotated and predicted by the Nt, Nr, Pfam, SwissProt, KOG, GO, and KEGG databases. Furthermore, differentially expressed genes (DEGs) were identified, and enrichment analysis based on GO and KEGG annotation was performed among the different developmental stages of the *R. pedestris* transcriptome library. Additionally, we obtained a different number of two molecular markers: single nucleotide polymorphisms (SNPs) and simple sequence repeats (SSRs). Finally, we summarized ten wing development-related signaling pathways, identified and characterized five full-length wing formation-related genes based on transcriptome annotation, and subsequently detected their expression patterns across all life stages of the bean bug. In summary, the results of this study may help to elucidate the formation mechanisms underlying wing polyphenism in hemipterous insects and establish a foundation for further research investigating plant–insect interactions and insect pest management in the agriculture industry.

## 2. Materials and Methods

### 2.1. Samples Collection and Rearing

Bean bug (*R. pedestris*) male and female adults were originally collected from an experimental field (39.1012° N, 117.1635° E) of Nankai University, Tianjin, P.R. China. The samples of all life stages obtained in this study originated from a single *R. pedestris* (monandrous) mating to obtain sufficient eggs, 1st- to 5th-instar nymphs and adults, which were reared on cowpea at 24 ± 2 °C with a photoperiod cycle of 14 h light/10 h dark and 70 ± 5% relative humidity (RH) in an RQH-25 intelligent artificial environment climate box (40 × 40 × 40 cm^3^) (JIUJIU, Jintan, China) at Nankai University, Tianjin, China [11]. Finally, all eggs, nymphs, and adults without abdomens were dissected and stored at −80 °C prior to RNA extraction.

### 2.2. RNA Extraction, Library Construction, and Illumina Sequencing

Total RNA from 20 eggs, 10 1st-instar nymphs, 10 2nd-instar nymphs, 10 3rd-instar nymphs, 10 4th-instar nymphs, 10 5th-instar nymphs and 10 adults (consisting of 5 females and 5 males) of *R. pedestris* was individually extracted according to the manufacturer’s instructions using TRIzol reagents (Invitrogen, Carlsbad, CA, USA), and the DNA contaminants and rRNA were removed by adding RNase-free DNase I (TaKaRa, Dalian, China) and Ribo-Minus (Invitrogen, Carlsbad, CA, USA), respectively. The degradation and contamination of the extracted RNA were monitored using gel electrophoresis on a 1% agarose gel. In addition, the quality and concentration of each RNA sample were quantified using the NanoPhotometer^®^ spectrophotometer (IMPLEN, Los Angeles, CA, USA) and the RNA Nano 6000 Assay Kit of the Agilent Bioanalyzer 2100 system (Agilent Technologies, Santa Clara, CA, USA).

Subsequently, sequencing libraries were generated using the NEBNext^®^ Ultra™ RNA Library Prep Kit for Illumina^®^ (NEB, Ipswich, MA, USA) according to the manufacturer’s recommendations, and index codes were added to attribute the sequences to each sample. Briefly, a total of 1.5 μg RNA per sample was used as input material for the RNA sample preparations. Furthermore, the mRNA purification, first- and second-strand cDNA synthesis, selection and purification of cDNA fragment-ligated adaptors, and amplification and purification of the final cDNA product (assessed on the Agilent Bioanalyzer 2100 system) from seven *R. pedestris* transcriptome libraries was performed as described in Wu et al. [12] Finally, after cluster generation on a cBot Cluster Generation System using the TruSeq PE Cluster Kit v3-cBot-HS (Illumina, San Diego, CA, USA), library preparations with 200 bp insert sizes were sequenced on an Illumina HiSeq 6000 platform to generate paired-end reads.

### 2.3. De novo Assembly and Functional Annotation of Transcriptomic Data

Raw data (raw reads) in Fasta format were processed to obtain clean data after removing reads with adapter or poly-N contamination, as well as low-quality reads. Moreover, the Q20, Q30, GC content, and sequence duplication level of the clean data were calculated [13,14]. Furthermore, we employed Benchmarking Universal Single Copy Orthologs (BUSCO) Version 2 to evaluate the quality and completeness of transcriptome assembly [15]. The clean reads were assembled using the Trinity program based on de novo results compared with the results obtained with different assemblers. Finally, the redundant clean reads were removed, and the clean reads were assembled to obtain unique transcript sequences (known as unigenes) [16].

In this study, the annotation of all the assembled unigenes was conducted against BLAST programs and ESTScan with the SwissProt, nonredundant protein sequences (Nr), NCBI nonredundant nucleotide sequences (Nt), Gene Ontology (GO), protein families (Pfam), Clusters of Eukaryotic Orthologous Groups (KOG), and KEGG Orthology (KO) databases [17]. All the above-mentioned annotations were performed with a cutoff e-value of <10^−5^.

### 2.4. Identification and Analysis of Differentially Expressed Genes

To calculate the expression changes of all unigenes between different transcriptome libraries, we applied the fragments per kilobase of transcripts per million mapped reads (PKFM) method to estimate the unigene expression levels with Bowtie2 software [18]. Furthermore, the differentially expressed genes (DEGs) were identified using the DEGseq (2010) R package, the *p*-value was adjusted using the *q*-value, and a *q*-value < 0.005 and |log2 (fold-change)|>1 was set as the threshold for significant differential expression based on Benjamini and Hochberg’s approaches [19,20]. In addition, a heatmap showing the differential unigenes of different developmental stages was produced via TIGR MultiExperiment Viewer. Finally, the GO enrichment and annotation of all DEGs was implemented using the GOseq R packages based on the Wallenius noncentral hypergeometric distribution, which included three categories: biological process, cellular component, and molecular function [21]. Additionally, we used KOBAS software to examine the statistical enrichment and annotation of differentially expressed genes in KEGG pathways to understand the high-level functions and utilities of the biological system [22]. In this study, we comparatively analyzed six comparison groups to illustrate the incremental changes in all developmental stages, including eggs vs. 1st instar nymphs (E vs. N1), 1st- vs. 2nd- instar nymphs (N1 vs. N2), 2nd- vs. 3rd- instar nymphs (N2 vs. N3), 3rd- vs. 4th- instar nymphs (N3 vs. N4), 4th- vs. 5th- instar nymphs (N4 vs. N5), and 5th- instar nymphs vs. adults (N5 vs. A).

### 2.5. Identification of Single Nucleotide Polymorphisms (SNPs) and Simple Sequence Repeats (SSRs)

In this study, to assess the assembled unigene quality and to identify potential SSR markers among all unigenes using the MISA tool, primers for each SSR were designed using Primer 5 [23,24]. Furthermore, SNPs within unigenes were identified across all developmental stages of the *R. pedestris* transcriptome using Picard tools (Version 1.41) and Samtools (Version 0.1.18), which were used to sort and remove duplicated reads and merge the bam alignment results of each sample. In addition, GATK3 software was used to perform SNP calling by filtering Raw vcf files [25].

### 2.6. Identification and Characterization of Wing Formation-Related Genes

To identify wing formation-related genes, we used tBLASTN searches to detect all putative genes from other insect species retrieved based on the NCBI database. The longest and complete length of transcripts was finally selected by BLAST search with an E-value cutoff of 1.0 e^−5^ and manually checked using the BLASTx program against the Nr database. Furthermore, the open reading frames (ORFs) of all putative wing formation genes were predicted by the ORF finder tool (https://www.ncbi.nlm.nih.gov/orffinder/, accessed on 10 November 2020). The domain distribution of all translated amino acid sequences was determined using the SMART server (http://smart.embl-heidelberg.de/, accessed on 10 November 2020), TMHMM 2.0 (http://www.cbs.dtu.dk/services/TMHMM/, accessed on 10 November 2020) and SignalP 4.0 (http://www.cbs.dtu.dk/services/SignalP/, accessed on 10 November 2020). Finally, the molecular weight and theoretical isoelectric point of all the proteins were estimated with the Expasy ProtParam tool (https://web.expasy.org/protparam/, accessed on 10 November 2020) [26,27].

### 2.7. Quantitative Real-Time PCR and Statistical Analysis

To analyze the expression changes of wing formation-related genes at different developmental stages, five unigenes were selected for further analysis by quantitative real-time PCR (qRT-PCR). First, the primer pairs of these genes were designed by Primer 5 software and are listed in Table S4. Furthermore, a SYBR Green real-time PCR assay was performed in an ABI StepOnePlus™ Real-Time PCR System (Applied Biosystems, USA) with the following PCR cycling conditions: 95 °C for 5 min followed by 40 cycles of 95 °C for 10 s, 60 °C for 20 s, and 72 °C for 20 s, and 75 °C for 5 s using cDNA samples, which were synthesized from total RNA by TransScript One-Step gDNA Removal and cDNA Synthesis SuperMix (TRANS, Beijing, China). The single melting curves for the EF-1α gene, as the endogenous reference, and all tested genes were calculated [11]. Finally, the relative expression of genes was analyzed by the 2^−ΔΔCT^ method, and the mean threshold cycle (Ct) was measured using three replicates for each gene [28].

Statistical analysis was performed using SPSS 17.0 software. The results of three independent experiments were expressed as the means ± SD. One-way analysis of variance (one-way ANOVA) was used to determine the significant difference in gene expression levels between different developmental stages of bean bug. *p*-values less than 0.05 were to be statistically significant.

## 3. Results and Discussion

### 3.1. Overview of Transcriptome Sequencing and Assembly

**Figure 1:**
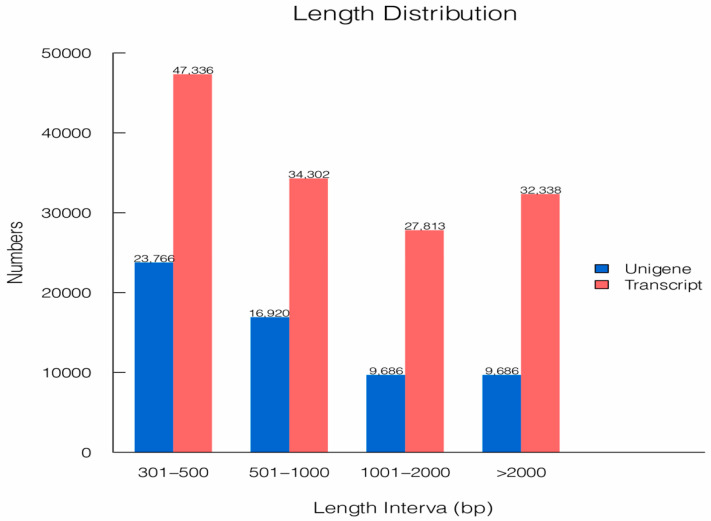
Length distribution of the unigenes and transcripts from the *R. pedestris* transcriptome library. The length intervals of unigenes and transcripts are presented on the *x*-axis, and the number is presented on the *y*-axis.

In this study, to explore the physiological changes occurring during *R. pedestris* development, seven transcriptome libraries were constructed and sequenced using the Illumina HiSeq6000 platform. As shown in Table 1, the transcriptome libraries of eggs (E), 1st-instar nymphs (N1), 2nd-instar nymphs (N2), 3rd-instar nymphs (N3), 4th-instar nymphs (N4), 5th-instar nymphs (N5) and adults (A) generated 44,548,968, 45,220,988, 44,478,730, 57,621,498, 40,174,406, 47,482,056 and 50,791,284 raw reads, respectively, and produced 43,520,034, 44,664,702, 43,384,364, 56,296,668, 39,511,894, 46,750,128, 49,596,154 clean reads, respectively, with a higher Q20 percentage and GC content after performing quality control, filtering data and removing low-quality or redundant reads. Furthermore, a total of 60,058 unigenes were obtained from 141,789 high-quality transcripts (with a 1469 bp average length and a 2642 bp N50 length) with an N50 length of 2126 bp and average length of 1199 bp (from 301 to 32,322 bp) using de novo assembly, and only 5.5% of unigenes were fragmented, while 3.8% were determined to be missing by BUSCO assessment (Table 2, Figure 1, Figure S1). Finally, the raw data, containing untrimmed data, have been deposited in the NCBI SRA database under the accession number PRJNA668857. Overall, we obtained abundant raw reads and assembled unigenes, higher than expected total number of genes found in other true bugs, which may establish a foundation for further functional annotation and classification analyses [29].

### 3.2. Functional Annotation and Classification of Unigenes

**Figure 2:**
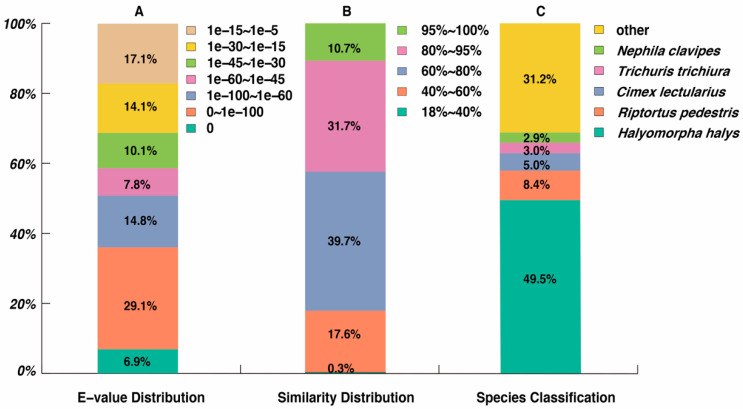
(**A**) E-values, (**B**) similarity, and (**C**) species distribution of *R. pedestris* transcriptome unigenes based on the Nr database by BLASTx search.

To analyze the putative function of unigenes, BLASTx tools (e-value < 1.0 × 10^−5^) were employed to search against the Nr, Nt, KO, SwissProt, Pfam, GO, and KOG databases. The results suggested that a total of 2,029 (3.48%) unigenes were annotated in all databases and that 25,881 (43.09%) were annotated in at least one database (Table 3). In addition, 19,449 unigenes (32.38%) had significant matches in the Nr database. A total of 68.8% of unigenes had significant homology (<1.0 × 10^−30^) to previously reported sequences by E-value distribution analysis (Figure 2A). The results of similarity distribution indicated that 82.7% of unigenes had more than 60% similarity to sequences against the Nr database (Figure 2B). Finally, the species distribution matched in the Nr database showed that 9,527 unigenes (49.5%) were similar to *Halyomorpha halys* followed by *R. pedestris* (1611 unigenes, 31.2%), *Cimex lectularius* (972 unigenes, 8.4%), and *Trichuris trichiura* (584 unigenes, 5%) (Figure 2C).

**Figure 3:**
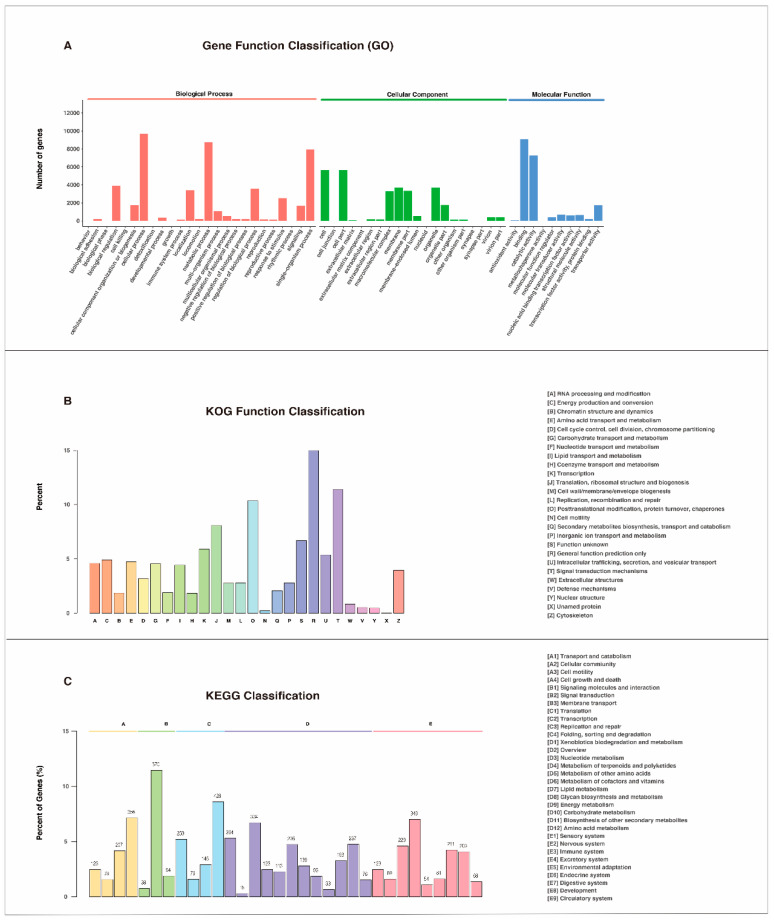
(**A**) GO (Gene Ontology) classification of the assembled unigenes in the *R. pedestris* transcriptome. Subcategories within each GO classification (biological process, cellular component, and molecular function) are also listed. The *y*-axis indicates the number of a specific category of genes in the main category, and the *x*-axis indicates gene numbers. (**B**) KOG (euKaryotic Ortholog Groups) classification of the assembled unigenes in the *R. pedestris* transcriptome. The name for each class definition is shown on the right. (**C**) KEGG (Kyoto Encyclopedia of Genes and Genomes) annotation analysis of the assembled unigenes in the *R. pedestris* transcriptome. A: Cellular Processes, B: Environmental Information Processing, C: Genetic Information Processing, D: Metabolism, E: Organismal Systems.

Furthermore, we characterized all assembled unigenes by GO, KOG, and KEGG databases to annotate their potential functions. First, all unigenes were selected for annotation with the GO database, suggesting that a total of 17,364 (28.91%) unigenes were mapped into three categories, including molecular function, cellular component, and biological process (Figure 3A). In brief, the top 3 biological processes were 9700 unigenes involved in cellular processes, 8742 unigenes involved in metabolic processes, and 7926 unigenes involved in single-organism processes; the top 3 molecular functions were 9095 unigenes involved in binding, 7285 unigenes involved in catalytic activity, and 1728 unigenes involved in transporter activity; and the top 3 cellular components were 5634 unigenes involved in cell parts, 3692 unigenes involved in membranes, and 3286 unigenes involved in macromolecular complexes (Table S1). Second, in total, the KOG classification divided 7594 (12.64%) unigenes into 26 functional categories (Figure 3B, Table S2). Among the functional classifications, the largest group was general function prediction (1137, 14.97%) followed by signal transduction mechanisms (865, 11.39%), posttranslational modification, protein turnover and chaperones (786, 10.35%), and translation, ribosomal structure, and biogenesis (612, 8.06%). Third, a total of 4973 unigenes (8.28%) were classified into five KEGG pathway functional categories, including cellular process (764 unigenes), environmental information processing (702 unigenes), genetic information processing (911 unigenes), metabolism (1826 unigenes), and organismal system (1127 unigenes), within 228 known KEGG pathways (Figure 3C, Table S3). In addition, signal transduction was the predominant group, including 216 unigenes, followed by translation (428 unigenes) and transport and catabolism (356 unigenes). Taken together, these data may elucidate the functions of unigene and provide valuable resources for further analysis of the *R. pedestris* transcriptome.

### 3.3. Detection of SSRs and SNPs

Simple sequence repeats (SSRs) are repeated sequences of 1 to 6 bp of DNA that have conserved flanking sequences and could be used for genomic mapping, evolutionary genetics, marker-assisted breeding, and marker-assisted selection in various species [25]. Therefore, we screened all unigenes of the *R. pedestris* transcriptome dataset to determine the nature and frequency of SSRs. In this research, a total of 35,158 SSRs from 60,058 unigenes were selected, and 7738 sequences containing more than one potential SSR were identified by searching for di-, tri-, tetra-, penta-, and hexanucleotide repeats (Table 4, Figure S2). Among all identified SSRs, the dinucleotide repeats numbered 6940, the majority of microsatellite repeat units followed by trinucleotide (1193), tetranucleotide (105), and hexanucleotide repeat motifs (5), which is similar to results of some previous reports on *Aphis aurantii* and *Adelphocoris suturalis* [29,30]. In addition, in the dinucleotide repeats, 4313 AT/AT were the primary types, and eight CG/CG were the minimum-respect sequences identified. The maximum and minimum trinucleotide repeats were AAT/ATT (751) and CCG/CGG (3), respectively. Moreover, to identify candidate SNP/INDEL positions in the *R. pedestris* transcriptome, we used SAMtools and VarScan software to align primitive sequences with all unigenes. As Table 5 shows, a total of 715,604 potential SNPs were identified from the seven transcriptome libraries, including 412,541 transition SNPs and 303,063 transversion SNPs. Furthermore, SNPs were typically located in the first (75,067), second (19,199), and third (92,709) codon positions.

These results provide large-scale genetic and genomic resources for research on the prevention of *R. pedestris* outbreaks. In keeping with other biological systems, we obtained 715,604 SNPs and 35,158 SSR variants in this study, which will be applicable in further pest control strategies in Chinese agriculture [29,31]. In summary, all candidate molecular markers selected in the *R. pedestris* transcriptome may provide more useful evidence for investigating genetic conservation, constructing genetic maps, and identifying genetic signatures of selection.

### 3.4. Identification and Annotation of Differentially Expressed Genes between Developmental Stages of R. pedestris

**Figure 4:**
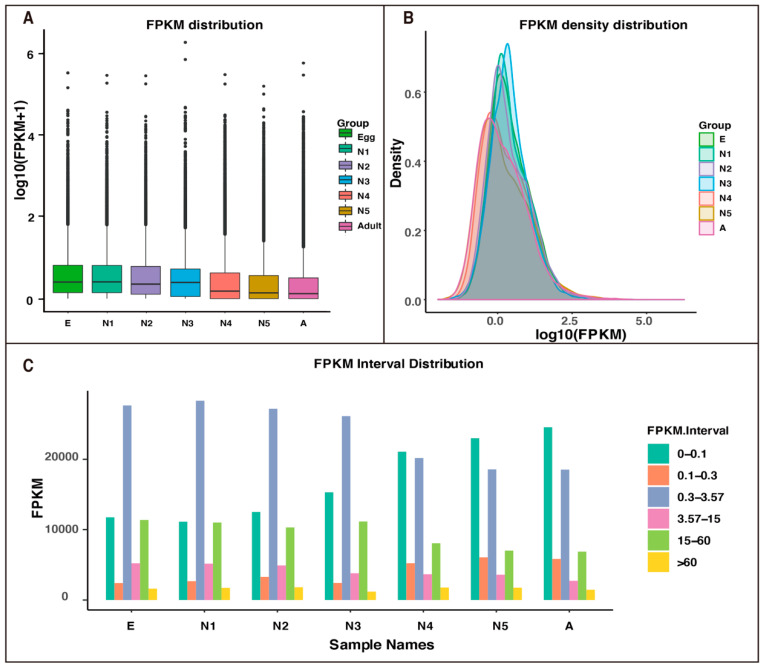
Expression levels of the *R. pedestris* unigenes in egg, 1st- to 5th-instar nymph, and adult by FPKM analysis. (**A**) The dispersing perspective. (**B**) The perspective of general distribution. (**C**) The interval distribution. E: eggs; N1: 1st-instar nymphs; N2: 2nd-instar nymphs; N3: 3rd-instar nymphs; N4: 4th-instar nymphs; N5: 5th-instar nymphs; A: adults.

To identify differentially expressed genes, a false discovery rate (FDR) < 0.001, an absolute fold-change > 2 and a *p*-value < 0.05 were utilized to calculate statistics for DEGs by using RSEM software within six comparison groups (E vs. N1, N1 vs. N2, N2 vs. N3, N3 vs. N4, N4 vs. N5, N5 vs. A). As a result, a number of read counts for each gene were obtained, and FPKM analysis was conducted accordingly. As shown in Figure 4, the FPKM distribution, interval, and density levels demonstrated that the gene expression quantity changed at different developmental stages of *R. pedestris*.

**Figure 5:**
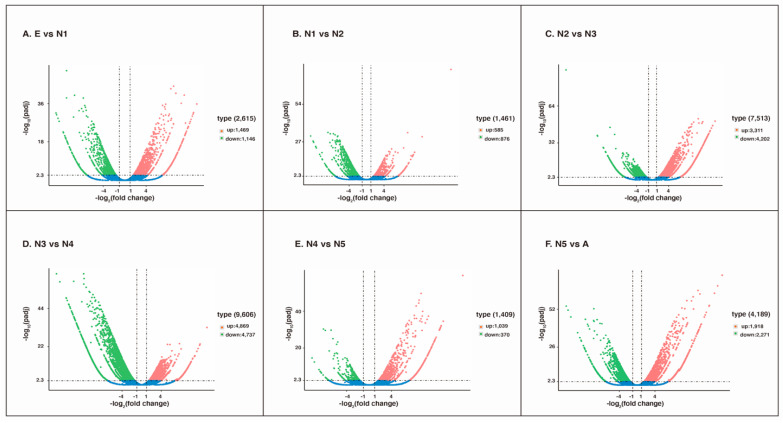
Volcano plots of the differentially expressed genes identified in the egg, 1st- to 5th-instar nymphs, and adult *R. pedestris* transcriptomes. (**A**) E vs. N1, (**B**) N1 vs. N2, (**C**) N2 vs. N3, (**D**) N3 vs. N4, (**E**) N4 vs. N5, (**F**) N5 vs. A. Splashes represent different genes. Blue splashes indicate genes with no significant differential expression, red splashes indicate significantly upregulated genes, and green splashes indicate significantly downregulated genes. E: eggs; N1: 1st-instar nymphs; N2: 2nd-instar nymphs; N3: 3rd-instar nymphs; N4: 4th-instar nymphs; N5: 5th-instar nymphs; A: adults.

**Figure 6:**
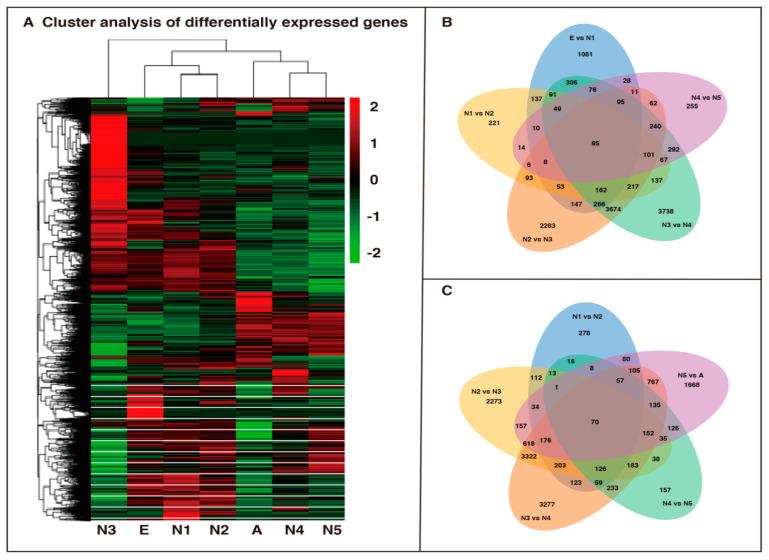
(**A**) Heatmap of differentially expressed genes (DEGs) among all unigenes based on FPKM units. Green indicates low expression, and red denotes high expression. (**B**,**C**) Venn diagram of the number of differentially expressed genes in the egg, 1st- to 5th- instar nymph, and adult *R. pedestris* transcriptomes. E: eggs; N1: 1st-instar nymphs; N2: 2nd-instar nymphs; N3: 3rd-instar nymphs; N4: 4th-instar nymphs; N5: 5th-instar nymphs; A: adults.

Overall, there were 2615 DEGs, with 1469 upregulated and 1146 downregulated unigenes, in the comparison between eggs and 1st-instar nymphs (Figure 5A). Similarly, between 1st- and 2nd-instar nymphs, 1461 unigenes showed significant expression changes, with 585 unigenes upregulated and 876 unigenes downregulated (Figure 5B). Next, the results of comparative analysis between 2nd- and 3rd-instar nymphs illustrated 7513 unigenes with significant expression changes, including 3311 upregulated unigenes and 4202 downregulated unigenes (Figure 5C). The maximum number of DEGs (9606 unigenes) was in the comparison group between 3rd- and 4th-instar nymphs, with 4869 being upregulated and 4737 downregulated (Figure 5D). However, the minimum DEG group contained 1409 unigenes with 1039 upregulated and 370 downregulated genes identified between 4th- and 5th-instar nymphs (Figure 5E). In the comparison between 5th-instar nymphs and adults, the expression profiles demonstrated that a total of 4189 unigenes had significant changes. Among these unigenes, 1918 unigenes were upregulated, and 2271 unigenes were downregulated (Figure 5F). In addition, we generated a heatmap using the Hcluster algorithm to visualize the expression patterns of all the unigenes in all developmental libraries (the color changes from red to green with decreasing expression) (Figure 6A). As shown in Figure 6B,C, different numbers of DEGs existed in different comparative groups, and only 95 and 70 DEGs existed in all five comparisons, respectively.

**Figure 7:**
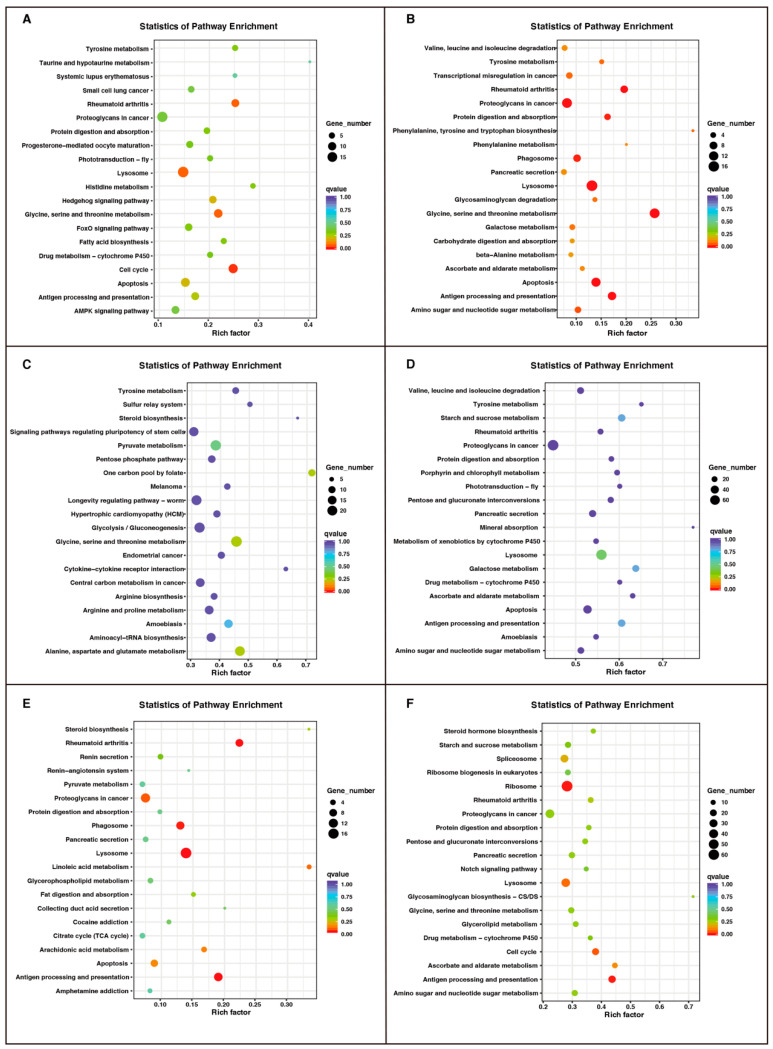
The top 20 significantly enriched KEGG pathways of the DEGs between different developmental stages of *R. pedestris*. (**A**) E vs. N1, (**B**) N1 vs. N2, (**C**) N2 vs. N3, (**D**) N3 vs. N4, (**E**) N4 vs. N5, (**F**) N5 vs. A. E: eggs; N1: 1st-instar nymphs; N2: 2nd-instar nymphs; N3: 3rd-instar nymphs; N4: 4th-instar nymphs; N5: 5th-instar nymphs; A: adults. The *x*-axis label shows the rich factor. The rich factor represents the number of DEGs/total number of genes in the KEGG pathway. The *y*-axis label shows the KEGG pathways. The color of the dots represents the *q*-value, and the size of the dot represents the number of DEGs enriched in the pathway.

Finally, to better determine the biological function of DEGs in six comparisons, we performed GO annotation and KEGG enrichment analysis to annotate the DEGs. Different numbers of up- and downregulated unigenes were significantly enriched GO terms, with biological process, cellular component, and molecular function, suggesting that the maximum and minimum numbers of DEGs were observed in the N2 vs. N3 and E vs. N1 comparisons, respectively (Figure S3, Table S5). As shown in Figure 7, we summarized the top 20 pathways in six comparison groups by KEGG enrichment. For example, the transcriptional changes were annotated in resistance and immune-related pathways, such as “Lysosome”, “Drug metabolism-cytochrome P450”, “Antigen processing and presentation” and “Apoptosis”, and metabolic-related pathways, including “Starch and sucrose metabolism”, “Ascorbate and aldarate metabolism”, “Porphyrin and chlorophyll metabolism”, “Tyrosine metabolism”, and “Amino sugar and nucleotide sugar metabolism”, in specific developmental stages, which might be closely associated with developmental process and survival activities. Taken together, the present study is the first report on all developmental life of *R. pedestris* that suggested the differential expression of the genes involved in various physiological and biochemical pathways, similar to other DEGs data, which could provide more evidence for interpreting the changes of wing developmental genes in hemipteran or other related species [5,29].

### 3.5. Identification and Analysis of Wing Formation-Related Signaling Pathways

To obtain further details on the wing formation of *R. pedestris* transcriptome libraries across all developmental stages based on the GO and KEGG databases, we selected a total of 426 unigenes in ten wing development-related signaling pathways, including the insulin signaling pathway, PI3K-Akt signaling pathway, mTOR signaling pathway, MAPK signaling pathway, JAK-STAT signaling pathway, Notch signaling pathway, Hedgehog signaling pathway, TGF-β signaling pathway, Hippo signaling pathway, and insect hormone biosynthesis with different numbers of DEGs in each library (Table 6). In brief, in comparison to N3 vs. N4, we detected maximum DEGs (125 unigenes) followed by N2 vs. N3 (88 unigenes), N5 vs. A (65 unigenes), E vs. N1 (27 unigenes), N4 vs. N5 (8 unigenes), and N1 vs. N2 (3 unigenes) from ten signaling pathways. The results of different DEGs being observed in six comparisons within ten signaling pathways might be associated with differences observed during tissue maturation and development in bean bug, including such processes as wing growth.

The insulin signaling pathway is an evolutionarily conserved nutrient-sensing pathway that participates in growth and development in metazoans and primarily activates the downstream PI3K-Akt signaling cascade by phosphorylated adapters [32,33,34,35]. For example, biological studies have elucidated the regulatory mechanism governing the insulin signaling pathway, which plays an important role in autonomously controlling body, organ, and cell size in *Drosophila* by encoding an insulin-like peptide to increase body size [36]. In a previous report, the migratory brown planthopper *Nilaparvata lugens* (Insecta, Hemiptera) was observed to possess *insulin receptor 1* (*InR1*), which leads to the long-winged morph if it activates the PI3K-Akt signaling cascade, and *insulin receptor 2* (*InR2*), which could negatively regulate the InR1–PI3K–Akt pathway to develop short-winged morphs in response to environmental or resource changes [37]. In addition, the three *insulin receptors* of the linden bug *Pyrrhocoris apterus* (Insecta, Hemiptera) were differentially silenced, and their participation in wing polymorphism control was confirmed [38]. Additionally, *mammalian target of rapamycin* (*mTOR*) activity is cell-autonomously stimulated by a series of extracellular stimulators, such as amino acids, glucose, and oxygen, to control growth and proliferation in species extending from invertebrates to vertebrates. In the mTOR signaling pathway in metazoans, *mTOR* activity was increased after an endocrine insulin signaling pathway triggered a conserved intracellular signaling cascade involving *PI3K* and *Akt* [32]. Based on these observations, we annotated and identified a total of 50, 61, and 13 DEGs in the insulin, PI3K-Akt, and mTOR signaling pathways, respectively. Notably, the N3 vs. N4 and N2 vs. N3 comparison groups had more DEGs than the other groups, suggesting that insulin receptors and downstream growth factors could play significant roles in wing formation during specific developmental periods in bean bug, similar to *N. lugens* and *Drosophila melanogaster*.

The Hippo pathway was first identified in *Drosophila* through the notable tissue overgrowth phenotypes resulting from mutations of Hippo or downstream factors, including the transcriptional coactivator *Yorkie*, as the nuclear effector, which combines with the TEAD family of DNA binding factors to activate transcription of cell growth and survival genes [39]. In fruit flies, two members of the Hippo signaling pathway, *Atg1* and *Acinus*, were subjected to targeted deletion in otherwise wild-type samples, which caused an increase in wing size and expression of target genes, and their overexpression inhibited growth [40]. In this study, we obtained 45 DEGs in the Hippo signaling pathway by KEGG enrichment. The results showed that the notable DEGs were primarily observed in three comparison groups, N2 vs. N3, N3 vs. N4 and N5 vs. A, implying that N3, N4, and adults were crucial stages in the transition from immature to mature wings in *R. pedestris*.

The Notch signaling pathway plays crucial roles in tissue development and homeostasis by regulating multiple biological processes, including cell fate determination, proliferation, and cycle progression. Upon binding to ligands, the *Notch receptor* generates the Notch intracellular domain (NICD) by a series of proteolytic cleavages, and the NICD subsequently translocates into the nucleus to regulate the expression of downstream target genes [41,42]. In a previous study, an ATPase, the *TER94* gene, played a novel role in development and could be involved in positively regulating the Notch signaling pathway by influencing the Notch target genes *wingless* and *cut* during wing margin formation in *D. melanogaster* [43]. Based on this result, a total of 27 DEGs were obtained in the Notch signaling pathway by GO and KEGG annotation. The results of the comparison between N3 vs. N4 and N5 vs. A groups indicated that the Notch signaling pathway could play an important role in these stages because the DEG numbers were greater than those of the other groups during *R. pedestris* wing development.

In addition, a total of 21 DEGs involved in insect hormone biosynthesis were identified by GO and KEGG enrichments in *R. pedestris* transcriptome libraries, indicating that N3, N4, and adults were significant developmental stages with maximal DEGs, which are in keeping with the results described above. Consistent with our data, a number of DEGs annotated in the biosynthesis of sesquiterpenoid juvenile hormone (JH) and ecdysteroid pathways were identified by BLAST search in *Phenacoccus solenopsis* to control the invasiveness of sap-sucking pests [5]. The hedgehog, MAPK, and JAK-STAT signaling pathways are also key members of conserved signaling pathways for evaluating such developmental defects in the *Drosophila* wing [44,45]. Hence, in this study, we also obtained different numbers of DEGs in these pathways, including maximal levels of DEG numbers in the N3 vs. N4 group, demonstrating that the three signaling pathways mentioned above play important roles in wing development for 4th-instar nymphs in *R. pedestris*. Moreover, the TGF-β signaling pathway is a hallmark of metazoan cell communication, exhibiting a suite of core TGF-β pathway proteins, including multiple ligands, at least three Type I receptors, one Type II receptor, and four or more Smad effector proteins that have transcription factor activity and exist in multiple larval tissues, including wing vein formation [46]. Our results showed that half of the DEGs (10 unigenes) annotated by the KEGG database were distributed in the comparison N3 vs. N4 group, similar to the above-mentioned pathways, indicating that these genes in the TGF-β signaling pathway could be involved in the wing formation of bean bug within a sophisticated regulatory network interaction with other pathways.

### 3.6. Identification and Expression Analysis of Wing Formation-Related Genes at Different Developmental Stages

**Figure 8:**
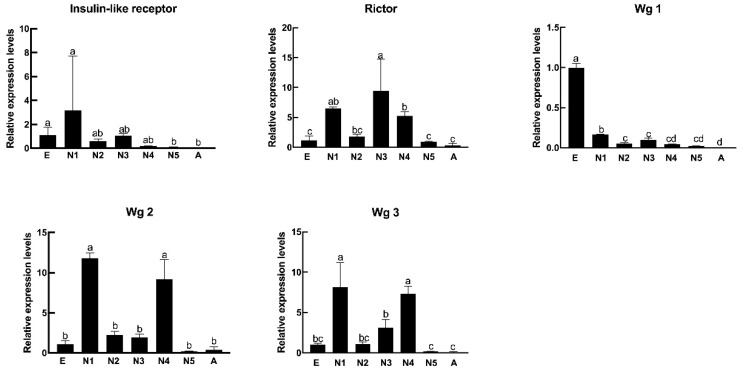
Expression profiles of wing formation-related genes at different developmental stages using qRT-PCR. E: eggs; N1: 1st-instar nymphs; N2: 2nd-instar nymphs; N3: 3rd-instar nymphs; N4: 4th-instar nymphs; N5: 5th-instar nymphs; A: adults. Each symbol and vertical bar represent the mean ± S.D. (*n* = 3). “a”, “b”, “c”, and “d” represent significant differences at *p*-values < 0.05.

To investigate and validate the molecular and expression characteristics of wing development-related genes across all life stages, a total of five unigenes were identified from the seven transcriptome libraries of *R. pedestris*, including the *insulin-like receptor* (*InR*), *rictor*, and *wingless* 1-3 (*wg* 1, *wg* 2, and *wg* 3) genes with similar tendency of up- or down-expression between qRT-PCR experiments and transcriptome analysis (Figure S4). First, an *InR* gene was found with a full-length open reading frame (ORF) of 4098 bp encoding 1365 amino acids (aa) (Table 7). Moreover, the qRT-PCR results showed that the *InR* gene was abundantly expressed in 1st-instar nymphs of *R. pedestris* (Figure 8). Previous data indicated that *insulin receptors* were divided into two families: cluster I, conserved in apical Holometabola for approximately 300 million years, and cluster II, present in the secreted decoy of *insulin receptor* in Muscomorpha due to ancestral duplication in a late ancestor of winged insects [38]. In this study, we selected a typical *insulin-like receptor* with two ligand-binding loops, furin-like cysteine-rich (Fu), three fibronectin type 3 (FN3), a single transmembrane (TM), and conserved tyrosine kinase (TyrKc) domains, and the expression patterns of this gene suggested that it could be involved in wing morph development and play significant roles in the development of 1st-instar nymphs of *R. pedestris*. Second, the cDNA sequence of *rictor* was identified from the *R. pedestris* transcriptome libraries. The ORF of *rictor* cDNA was determined to be 4320 bp and to encode a of 1,439 aa polypeptide with a theoretical molecular mass of 159.97 kDa and an isoelectric point of 6.42 (Table 7). Furthermore, as shown in Figure 8, quantitative real-time PCR was performed to determine its expression pattern during all developmental stages of *R. pedestris*, suggesting that an increasing expression level was estimated from eggs to 4th-instar nymphs, and the *rictor* gene was maximally expressed in 3rd-instar nymphs. *Rictor* (*rapamycin-insensitive companion of mTOR*) is a key member of the mTOR signaling pathway that is beneficial to assembly and promotes the activity of *mechanistic target of rapamycin complex 2* (*mTORC2*), which primarily participates in cytoskeletal organization, cell migration, modulation of cell cycle progression, and control of cell survival [47]. In addition, *rictor* mutants result in reduced body weight and shrinking eyes and wings in a *Drosophila* model system [48]. In this study, the *R. pedestris rictor* gene was ubiquitously expressed in all life stages but was primarily expressed in 1st-, 3rd- and 4th-instar nymphs, indicating that this gene could play a crucial role in wing development at specific life stages by activating and phosphorylating key factors in the mTOR or PI3K–Akt signaling pathways, such as *AKT* and *protein kinase C*.

Finally, we further selected three full-length *wingless* genes from the *R. pedestris* transcriptome based on the annotation information. All cDNA and protein sequence information regarding the *wingless* genes is listed in Table 7, showing that the ORF lengths of the three *wingless* genes are 1059 bp, 1038 bp, and 1173 bp, encoding 352 aa, 335 aa and 390 aa proteins, respectively. Additionally, the molecular weights (MWs) of the proteins ranged from 43.40 kDa to 38.84 kDa, and the theoretical pI values varied from 9.54 to 8.91. As shown in Figure 8, the expression of three *wingless* genes appeared to change across different developmental stages. In brief, the temporal expression of *wg* 1 was upregulated and reached a maximum in eggs of *R. pedestris* and was subsequently downregulated from 1st-instar nymphs to adults of *R. pedestris*. The *wg* 2 mRNA transcripts were significantly increased and reached the maximum expression level in 1st-instar nymphs, and it was also primarily expressed in 4th-instar nymphs of *R. pedestris*. Moreover, in keeping with the *wg* 2 expression, *wg* 3 was also primarily distributed in 1st- and 4th-instar nymphs of *R. pedestris*. From nematodes to mammals, the wnt signaling pathway includes a large family of cysteine-rich secreted glycoproteins that are involved in controlling animal development. The *wnt* genes exhibit sequence homology from *wnt* in the mouse to *wingless* in *Drosophila*. In holometabolous insects, such as flies and butterflies, accurate patterning and development are regulated by a series of gene expression patterns in tissues at specific developmental stages [49]. For example, *wingless* is a morphogen that acts as a short-range inducer and a long-range organizer across development in *Drosophila* and participates in patterning of wing discs followed by specification of wing margin-specific patterns [50]. Taken together, the various expression profiles of *wingless* genes demonstrated that the specific regulatory mechanisms of *wingless* members involved in the wnt signaling pathway were active in hemimetabolous insects, especially bean bug.

## 4. Conclusions

In this study, we obtained a total of 60,058 unigenes assembled and annotated by raw reads and public databases, respectively, from transcriptome libraries obtained at all life stages of *R. pedestris*. Furthermore, comparative transcriptomic data among all developmental stages enabled the detection of numerous differentially expressed genes and the characterization of ten signaling pathways involved in developmental processes and wing formation in bean bug, suggesting that 3rd- vs. 4th-instar nymphs possessed major DEGs in ten wing development-related signaling pathways. In addition, 35,158 SSRs and 715,604 SNPs were selected from all these transcriptomes. Finally, the molecular and expression features of five wing formation-related genes were analyzed at the various developmental stages of *R. pedestris*. Overall, our results may establish a foundation for further research investigating the developmental process at the molecular level and for the application of improved biological control agents in *R. pedestris*.

## Figures and Tables

**Table 1:** Summary of output statistics from all developmental stages of *R. pedestris*.

Sample	Raw Reads (bp)	Clean Reads (bp)	Clean Bases (G)	Error Rate	Q20 (%)	Q30 (%)	GC (%)
E	44,548,968	43,520,034	6.53	0.03	97.2	92.24	36.18
N1	45,220,988	44,664,702	6.7	0.03	97.6	93.22	35.97
N2	44,478,730	43,384,364	6.51	0.03	97.62	93.23	37.05
N3	57,621,498	56,296,668	8.44	0.03	97.74	93.23	33.46
N4	40,174,406	39,511,894	5.93	0.03	97.71	93.49	39.92
N5	47,482,056	46,750,128	7.01	0.03	97.93	93.94	40.3
A	50,791,284	49,596,154	7.44	0.03	97.81	93.69	39.68

**Table 2:** Summary of assembled transcripts and unigenes.

	Maximum Length (bp)	Minimum Length (bp)	Average Length (bp)	N50 (bp)	Total Number	Total Nucleotides (nt)
Transcripts	32,322	301	1469	2642	141,789	208,288,687
Unigenes	32,322	301	1199	2126	60,058	71,994,954

**Table 3:** Statistics of functional annotation from *R. pedestris* unigenes.

	Number of Unigenes	Percentage (%)
Annotated in NR	19,449	32.38
Annotated in NT	11,193	18.63
Annotated in KO	4973	8.28
Annotated in SwissProt	14,374	23.93
Annotated in PFAM	17,364	28.91
Annotated in GO	17,364	28.91
Annotated in KOG	7594	12.64
Annotated in all Databases	2091	3.48
Annotated in at least one Database	25,881	43.09

**Table 4:** Statistics of simple sequence repeats (SSRs) detection in *R. pedestris* transcriptome.

Items	Numbers
Total number of sequences examined	60,058
Total size of examined sequences (bp)	71,994,954
Total number of identified SSRs	35,158
Number of SSR containing sequences	20,778
Number of sequences containing more than one SSR	7738
Number of SSRs present in compound formation	3251

**Table 5:** Summary of single nucleotide polymorphism (SNP) type from *R. pedestris* transcriptome.

Type	Count	Frequency Per kb
Transition		
C/T	205,816	2.86
A/G	206,725	2.87
Transversion		
A/T	126,216	1.75
A/C	65,010	0.9
T/G	65,248	0.91
C/G	46,589	0.65
Total	715,604	9.94
SNP position in codon		
First	75,070	
Second	19,199	
Third	92,709	

**Table 6:** Statistics of DGEs of wing formation-related pathways in *R. pedestris* transcriptome based on KEGG enrichment.

	Egg vs. N1	N1 vs. N2	N2 vs. N3	N3 vs. N4	N4 vs. N5	N5 vs. Adult	Total DEGs
Insulin signaling pathway	5	1	12	19	4	9	50
PI3K-Akt signaling pathway	5	0	19	25	1	11	61
mTOR signaling pathway	0	0	6	7	0	0	13
MAPK signaling pathway	3	0	13	19	0	10	45
JAK-STAT signaling pathway	2	0	6	6	0	1	15
Notch signaling pathway	2	0	6	9	1	9	27
Hedgehog signaling pathway	5	1	5	12	1	8	32
TGF-β signaling pathway	1	0	6	10	1	2	20
Hippo signaling pathway	2	0	15	19	0	9	45
Insect Hormone Biosynthesis	2	1	6	6	0	6	21
Summary	27	3	94	134	8	65	329

**Table 7:** The cDNA and protein features of seven wing developmental genes from *R. pedestris* transcriptome.

	Insulin-Like Receptor	Rapamycin-Insensitive Companion of mTOR (Rictor)	Wingless 1 (wg 1)	Wingless 2 (wg 2)	Wingless 3 (wg 3)
ORF length (bp)	4098	4320	1059	1008	1173
Amino acids length	1365	1439	352	335	390
Weight (kDa)	155.13	159.97	39.03	38.84	43.40
Theoretical pI	6.19	6.42	9.00	8.91	9.54
Formula	C_6871_H_10731_N_1873_O_208_0S_71_	C_7121_H_11328_N_1944_O_2135_S_51_	C_1684_H_2658_N_508_O_501_S_31_	C_1668_H_2677_N_501_O_498_S_35_	C_1873_H_2968_N_584_O_542_S_33_
NCBI accession number	MW280220	MW280221	MW280222	MW280223	MW280224

## Data Availability

Data is contained within the article or Supplementary Material.

## Supplementary Materials

The following are available online at https://www.mdpi.com/2075-4450/12/3/226/s1, Table S1. GO classification in the *R. pedestris* transcriptome. Table S2. KOG classification in the *R. pedestris* transcriptome. Table S3. KEGG classification in the *R. pedestris* transcriptome. Table S4. Primers for quantitative real-time PCR analysis in this study. Table S5. GO classification of DEGs in the *R. pedestris* transcriptome. Figure S1. The evaluation of the accuracy and completeness of the *R. pedestris* transcriptome. Figure S2. Scattergram of simple sequence repeats (SSRs) detected in the *R. pedestris* transcriptome unigenes. Figure S3. Gene ontology (GO) analysis of DEGs between different developmental stages in *R. pedestris*. (A) E vs. N1, (B) N1 vs. N2, (C) N2 vs. N3, (D) N3 vs. N4, (E) N4 vs. N5, (F) N5 vs. A. Unigenes were summarized into three main categories, namely, biological process (BP), cellular component (CC), and molecular function (MF). The *x*-axis label denotes the number of unigenes, whereas the *y*-axis label represents the unigenes’ respective GO terms. E: eggs; N1: 1st-instar nymphs; N2: 2nd-instar nymphs; N3: 3rd-instar nymphs; N4: 4th-instar nymphs; N5: 5th-instar nymphs; A: adults. Figure S4. Expression profiles validation of five wing formation-related genes between qPCR (blue charts) and transcriptome sequencing (red lines) in *R. pedestris*. E: eggs; N1: 1st-instar nymphs; N2: 2nd-instar nymphs; N3: 3rd-instar nymphs; N4: 4th-instar nymphs; N5: 5th-instar nymphs; A: adults. The red lines showed the expression levels of above genes in different developmental stages detected by FPKM method. The correlation coefficient between the qPCR data and the transcriptome data are shown in each group.
