# Supplementary figures and images for: Comparative Transcriptomic Analysis of Riptortus pedestris (Hemiptera: Alydidae) to Characterize Wing Formation across All Developmental Stages
Source: Insects. 2021 Mar 5;12(3):226. doi: 10.3390/insects12030226 (PMC7999114; doi:10.3390/insects12030226)

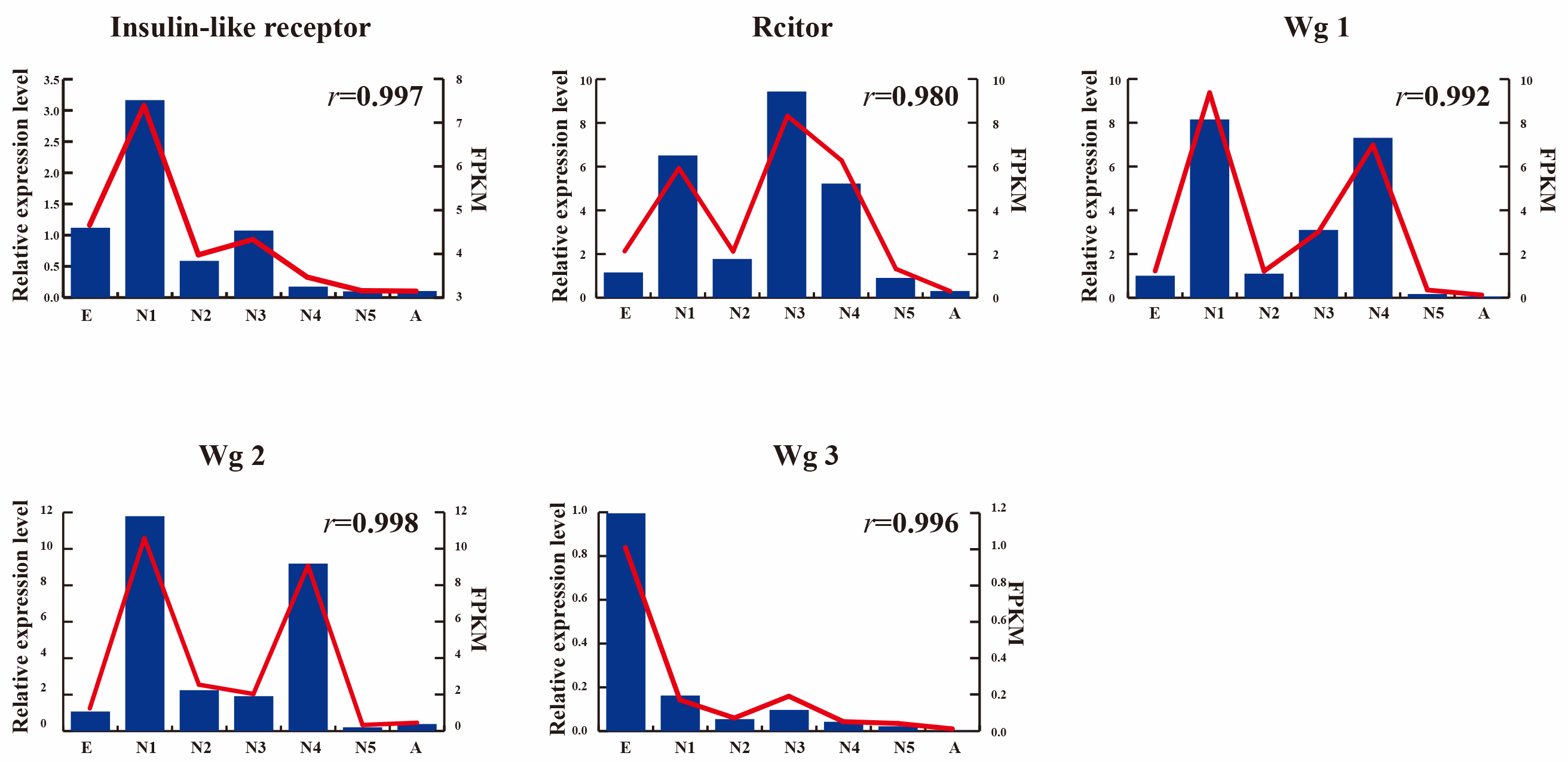

Supplement: Supplementary file 1 [file insects-12-00226-s001.zip › Supplementary material/Figure S4.tif]

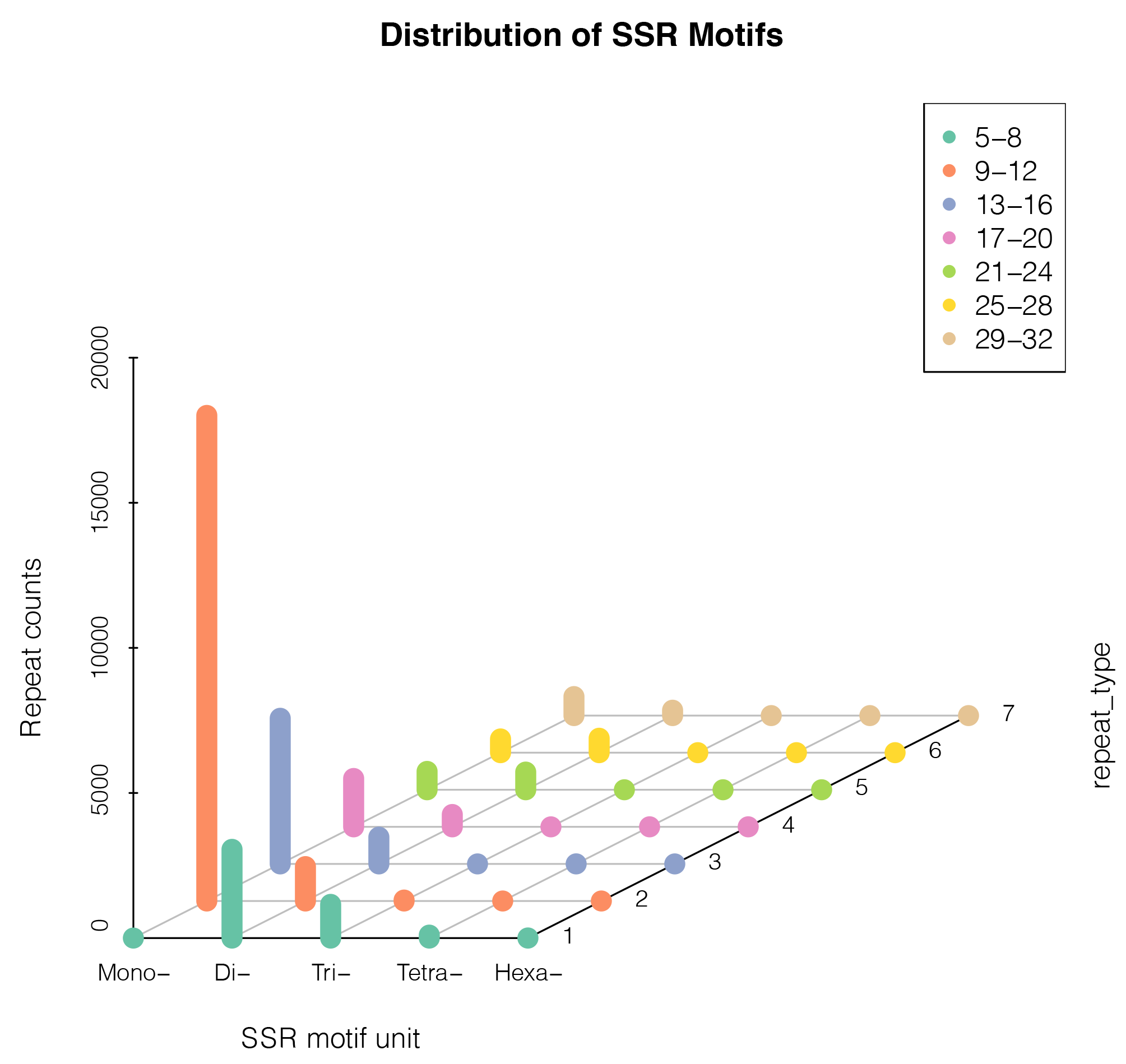

Supplement: Supplementary file 1 [file insects-12-00226-s001.zip › Supplementary material/Figure S2.tif]

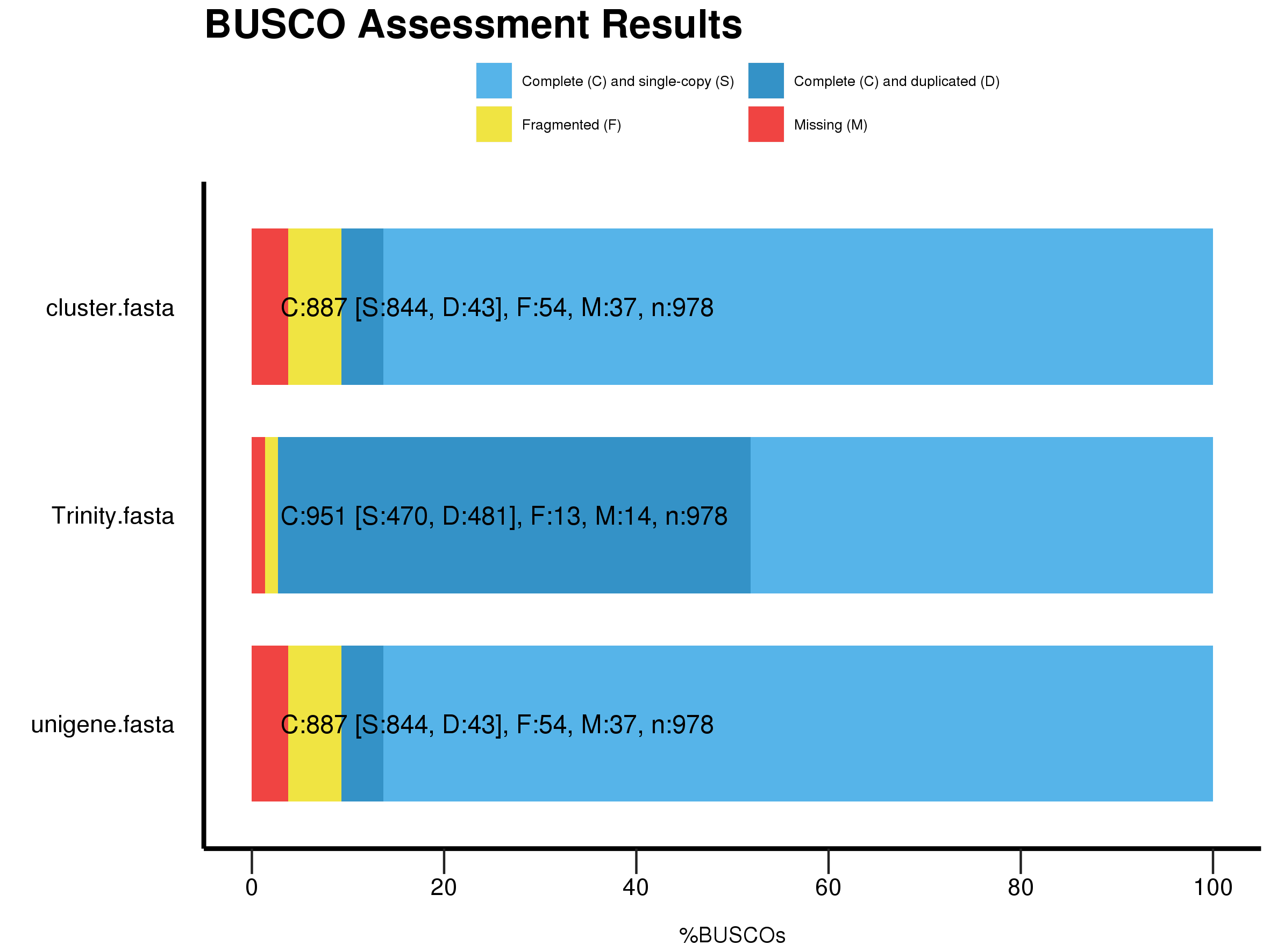

Supplement: Supplementary file 1 [file insects-12-00226-s001.zip › Supplementary material/Figure S1.tif]
