# Supplementary material for: Comparative Transcriptomic Analysis of Riptortus pedestris (Hemiptera: Alydidae) to Characterize Wing Formation across All Developmental Stages
Source: Insects. 2021 Mar 5;12(3):226. doi: 10.3390/insects12030226 (PMC7999114; doi:10.3390/insects12030226)
Supplement: Supplementary file 1 [file insects-12-00226-s001.zip › Supplementary material/Table S4.doc]

Table S4. Primers for quantitative real-time PCR in this study.

| Unigene ID | Primer | Primer sequence (5’- 3’) |
| --- | --- | --- |
| insulin receptor | Sense | AAATACAAATACGAGGAGGA |
| insulin receptor | Antisense | ATACTATTGGGAATGAACGA |
| rictor | Sense | CCTTGGAGGATTTCGTTA |
| rictor | Antisense | ATGGGACAGGTGGCTT |
| wg 1 | Sense | AGTTTACGGTTACAGGTT |
| wg 1 | Antisense | CTTCATTATTGTGGAGGT |
| wg 2 | Sense | TTAGAAGATTCGCCAGAT |
| wg 2 | Antisense | TAACATTACAGCACCACA |
| wg 3 | Sense | CAAACCGCCTGGCTCAAA |
| wg 3 | Antisense | CGCAACCGTCCACTCCTA |
| EF-1α | Sense | AATCGACTGAGCAGAACCCT |
| EF-1α | Antisense | GAGGTGGGAATTCTTGGAAA |
